# Acclimation and interaction between drought and elevated UV-B in *A. thaliana*: Differences in response over treatment, recovery and reproduction

**DOI:** 10.1002/ece3.387

**Published:** 2012-09-27

**Authors:** David Comont, Ana Winters, Dylan Gwynn-Jones

**Affiliations:** Institute of Biological Environmental and Rural Sciences, Aberystwyth UniversityCeredigion, SY23 3DA, WALES, UK

**Keywords:** Acclimation, drought, interaction, phenology, reproduction, UV-B

## Abstract

Here, a factorial experiment was used to investigate the interactive effects of a UV-B episode and concurrent progressive drought on the growth, chemistry, and reproductive success of *A. thaliana*. Both drought and UV-B negatively affected rosette growth, although UV-B had the greater effect. Acclimation to UV-B involved adjustment of leaf morphology, while drought induced accumulation of soluble sugars and phenolics. All plants recovered from treatments, but the cost of recovery was a developmental delay resulting in alteration in phenological timings. Combined treatments interacted causing additive negative effects on growth following exposure. This may be linked with inhibition of soluble sugar accumulation by UV-B, restricting the capacity for osmotic adjustment in response to drought. Following cessation of treatments, relative growth rate (RGR) and net assimilation rate (NAR) were significantly stimulated in plants treated with combined drought and UV-B. This interaction alleviated subsequent impacts of elevated UV-B on silique yield and reproductive timings. This study demonstrates the potential for interaction between these two common environmental factors. Furthermore, it shows the changeable nature of these interactions over the course of exposure and recovery through to reproduction, highlighting the need for sustained assessment of such interactions over a plant's lifecycle.

## Introduction

*Arabidopsis thaliana* is an internationally recognized model species with applications in a diverse range of fields from genetics and cell biology to physiological, developmental, and evolutionary biology (Mitchell-Olds 2001; Pigliucci 2002). As a result, this species is important in the development of our understanding of plant responses to abiotic stimuli such as Ultraviolet-B (UV-B) radiation. Solar UV-B radiation (290–320 nm) comprises approximately 1.5% of the solar irradiance reaching Earth (Hollosy 2002), with lower wavelengths absorbed within the atmosphere. Ozone depletion has caused UV-B levels in the Northern hemisphere to increase significantly over the last 30 years (Herman, 2010), and so many studies have investigated UV-B responses in relation to ozone depletion example (Searles et al. 2001; Rozema et al. 2005). Ozone depletion has now been limited (Austin et al. 2010); however, responses to UV-B remain important for study due to the substantial variation in ambient UV-B across terrestrial ecosystems (Paul and Gwynn-Jones 2003), and the likelihood of further changes in UV-B irradiance with climatic change (Ballaré et al. 2011).

UV-B is known to result in a range of photomorphogenic responses; for example, Kim et al. (1998) found UV-B to inhibit hypoctyl elongation in *A. thaliana*, whereas Hectors et al. (2007) identified a reduction in *A. thaliana* rosette area by UV-B. This has been linked to a reduction in cell expansion by UV-B, affecting leaf development and morphology (Hectors et al. 2010). Under higher dose rates, UV-B is known to act as an oxidative stress (Landry et al. 1995), and causes a further range of characteristic plant responses including accumulation of UV-B absorbing chemicals (Landry et al. 1995; Bashandy et al. 2009), and alteration in morphology such as increased leaf thickness and trichome production (Tattini et al. 2000; Liu et al. 2005). Recovery following UV-B exposure is known to involve a further set of responses including allocation of resources to maximize leaf area (Stephanou and Manetas 1997), and upregulation of pathways involved in repair of UV-B-induced DNA damage (Britt 2004). However, under field conditions, responses to UV-B radiation are often less pronounced (Ballaré et al. 2011). This may be due to the influence of other co-occurring abiotic factors (Mittler 2006), as it has now been shown that responses to UV-B radiation may interact with other environmental stimuli (Caldwell et al. 2007).

One particular source of UV-B interaction may be drought. Kilian et al. (2007) identified a substantial overlap in gene expression of *A. thaliana* in response to both UV-B and drought. Similarly, Schmidt et al. (2000) identified ameliorative effects of combined drought and UV-B on maintenance of leaf relative water content in *A. thaliana* (Schmidt et al. 2000). Nevertheless, whilst drought and UV-B have been shown to interact, responses are not always consistent between studies. Sangtarash et al. (2009) found combined drought and UV-B to have an additive effect upon reduction of leaf area and biomass production in *Stellaria longipes*. Similarly Tian and Lei (2007) found the combined factors to have an additive negative effect on the growth of *Triticum aestivum* seedlings, possibly related to combined oxidative damage. Conversely, Alexieva et al. (2001) found that *Pisum sativum* and *Triticum aestivum* exposed to combined drought and UV-B gained a greater total biomass than plants exposed to UV-B alone. Similarly, He et al. (2011) found that pre-application of drought caused increased tolerance to UV-B and vice versa in *triticum aestivum*, suggesting some cross-tolerance between these factors. Such contrasting responses highlight the capacity for interaction between drought and UV-B, but demonstrate the need for further study of these interactive responses.

One currently under-researched aspect of response to both UV-B and drought is their role as regulators of plant phenology and reproductive success. Maintenance of reproduction following stress is often accompanied by changes in phenology, (Pigliucci and Schlichting 1995; Brun et al. 2003). For example Nord and Lynch (2008) found a delay in flower production in *A. thaliana* in response to phosphorous deficiency, whereas Dorn et al. (2000) identified a phenological delay caused by shading. Feng et al. (2007) found UV-B and drought to have opposite effects on phenological timings in *Triticum aestivum*; therefore, there may be potential for interactive effects of drought and UV-B on plant phenology. Both drought and UV-B are further known to result in reproductive costs; for example, drought caused a significant reduction in yield of *Oryza sativa* (Boonjung and Fukai 1996), whereas UV-B caused a reduction in seed yield and an increase in the number of unseeded pods in *Glycine max* (Chimphango et al. 2007). Such loss of productivity can have considerable consequences, with drought alone estimated to cause $20 billion (US) in agricultural losses between 1980 and 2004 (Mittler 2006). However, water deficit alleviated UV-B-induced decreases in yield in *Glycine max*, causing a greater reproductive success than at elevated UV-B alone (Sullivan and Teramura 1990). It has therefore been suggested that drought and UV-B combined might result in an improved reproductive success in comparison with either factor in isolation.

Drought and UV-B co-occur under conditions of high solar irradiance and low precipitation during periods when atmospheric pressure is high resulting in cloudless or heat-wave events, which are predicted to increase in frequency and intensity over the coming century (Ganguly et al. 2009). Such conditions are generally episodic, and last from days to weeks. The current study was designed to investigate such an ‘episode’ of co-occurring UV-B and drought under controlled glasshouse conditions, and subsequently identify if interactive effects are maintained during phenological development through to reproduction. We assessed plant growth and metabolite responses using a combination of classical growth analysis (Hunt et al. 2002), assessment of sugars, and methanol-soluble phenolics via high-performance liquid chromatography (HPLC), and microscopy of leaf characteristics. It is hypothesized that both drought and UV-B will negatively affect plant growth and reproductive success. However, these factors will interact counteractively during treatment or recovery to facilitate maintenance of reproductive parameters.

## Materials and Methods

A factorial experiment was designed to investigate the interaction between co-occurring drought and UV-B over a short episode such as may accompany a cloudless period of high pressure-dictated weather or heat-wave event (Ganguly et al. 2009). Interactive responses were further assessed over the remainder of the plants lifecycle, to identify the capacity for interaction between these factors during recovery from treatments, and to identify the effects of a combined UV-B and drought episode on plant phenology and reproductive success. *Arabidopsis thaliana* (Col-0) was chosen for this study as it is one of the most frequently used ecotypes for experimentation, and a previous study identified a characteristic set of growth and reproductive responses to prolonged UV-B in this ecotype (Lake et al. 2009).

### Plant material and growth conditions

Wild type *Arabidopsis thaliana* (Col-0) plants were grown from seed in Levingtons F2 compost in individual 5 × 5 × 5 cm (length × width × depth) pots for 5 weeks within a controlled environment cabinet. Day/night temperatures were 22/18°C with a light intensity of 140 μmol/m^2^/s over an 8-h photoperiod. Five-week-old plants were transferred to a glasshouse where they remained throughout the experiment. Glasshouse temperature was not controlled, but remained on average 14.7 ± 4.1°C, and supplementary PAR (200 ± 10 μmol/m^2^/s) was provided throughout by banks of high-power sodium lamps. Mean daytime PAR was approximately 1000–1500 μmol/m^2^/s; however, this varied greatly both within and between days due to fluctuation in ambient solar conditions.

**Figure 1:**
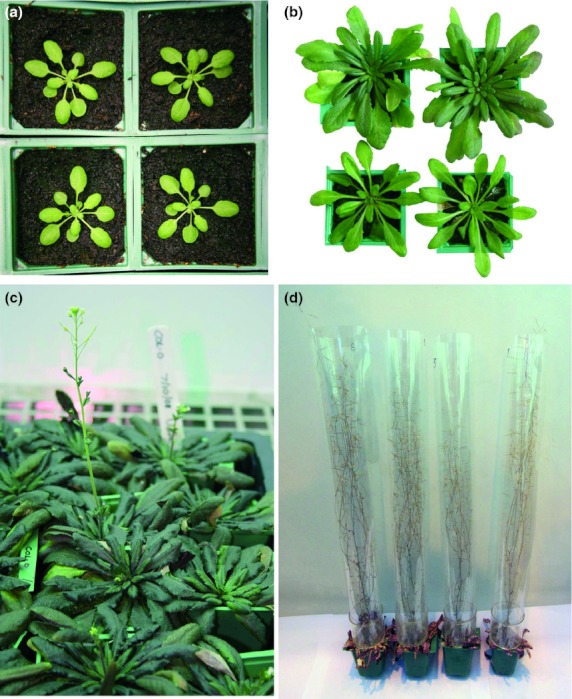
Representative *A. thaliana* plants over the course of the lifecycle studied. (a) Before treatment, (b) following treatment and subsequent recovery, (c) Rosettes at onset of reproduction, and (d) Plants with mature floral stems, shown with aracon tubes fitted to aid seed collection.

We simulated UV-B conditions typical of cloudless spring/summer periods in the UK with subsequent drought exposure, which often lags days later. The experimental conditions consisted of two watering regimes (well watered vs. droughted), and two UV-B regimes (elevated vs. control UV-B) providing a two-way factorial design. An initial T = 0 harvest of six plants was performed before experimental treatments were started. Following this, 14 days after the treatments were initiated, harvest 1 (post treatment) was performed. Plants were allowed to recover for a further period of 14 days before harvest 2 (post recovery). At each harvest, six plants per treatment were assessed for growth measurements (*n* = 6) with further six plants per treatment flash-frozen in liquid Nitrogen for chemical analyses. After the final harvest, 20 plants from each treatment were maintained and allowed to reproduce within individual aracon tubes (Betatech, Gent, Belgium) (Fig. 1).

### Experimental treatments

**Figure 2:**
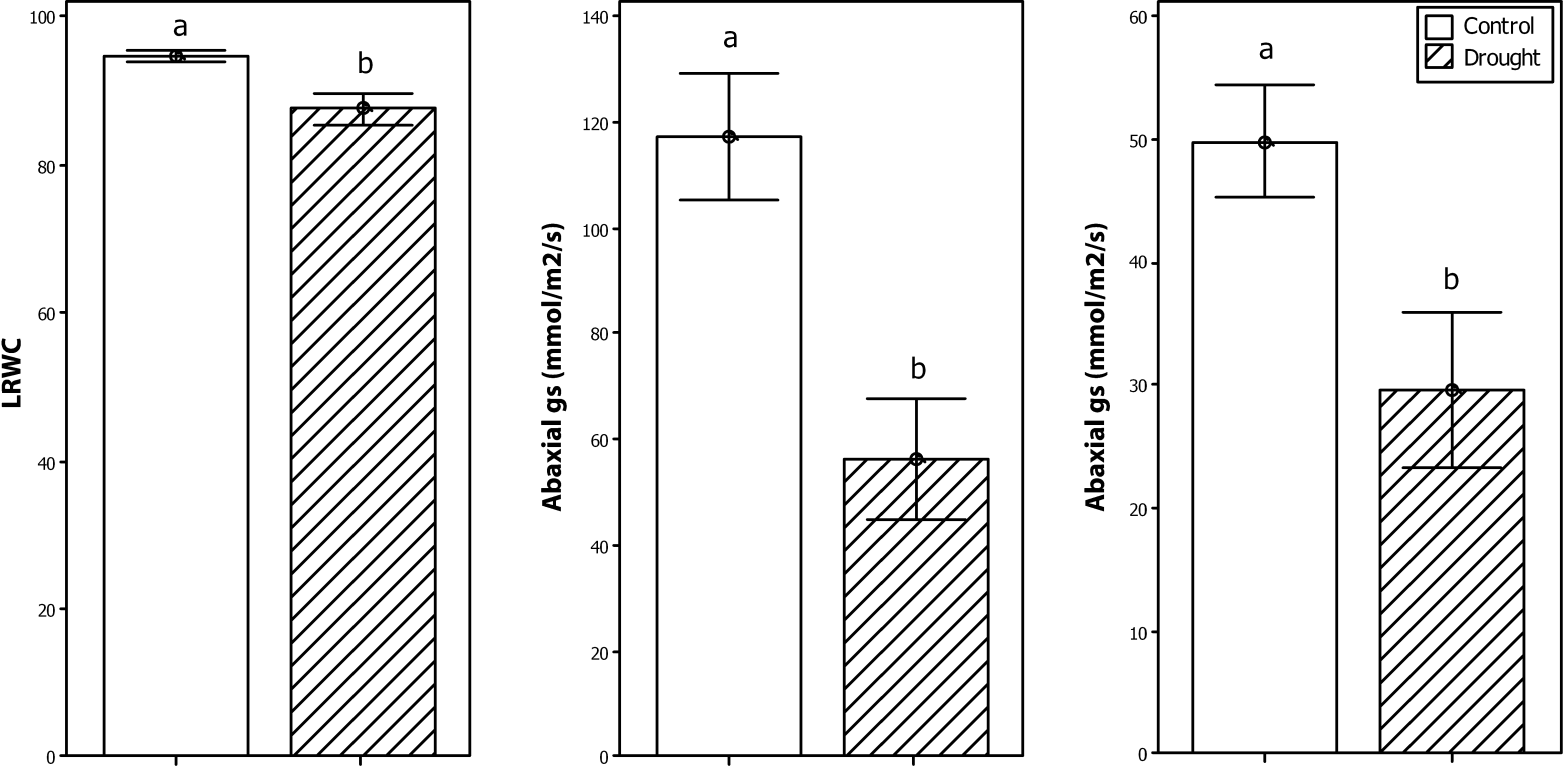
The effect of a 14-day drought treatment on leaf relative water content (LRWC), and stomatal conductance (g_s_) of the abaxial and adaxial leaf surface. Bars show the mean (± SE), and letters denote significant differences following a one-way ANOVA coupled with a Tukey's multiple range test. Stomatal conductance (g_s_) was measured using a porometer (AP4, Delta-T devices, Cambridge, UK). Leaf relative water content (LRWC) was assessed from leaf disks as per Machado and Paulsen (2001). In each case, three randomly chosen fully extended leaves were measured per plant from six plants per treatment.

Some studies manipulate water availability directly to provide instantaneous water deficit, which is sustained at a particular intensity, for example, Wang et al. (2008). However, under field conditions, drought is often progressive, building in intensity over time. The current study was designed to provide such a progressive drought treatment by withholding water over 14 days, while the controls were provided with water regularly. A longer drought treatment (19 days) was trialed, but was found to cause plant mortality. Drought intensity was monitored throughout the experiment using tensiometers (SKTM 650, Skye Instruments, Llandrindod Wells, UK), (Appendix 1, a). The drought treatment provided was still relatively ‘mild’ by the end of the treatment period, but was sufficient to cause visible wilting with concomitant stomatal closure and reduced leaf relative water content (LRWC) (Fig. 2).

Elevated UV-B was provided using Q-Panel 313 lamps (Q-Panel, Cleveland, Ohio, USA) wrapped in cellulose diacetate (0.13 mm, Courtaulds, Derby, UK) to attenuate UV-C. The daily UV-B_BE_ dose was 5.47 kJ/m^2^/day, measured using an EPP2000 Fibre Optic Spectroradiometer with an F400 UV/VIS fiber optic cable (StellarNet Inc, Tampa, Florida, USA) and weighted using the plant damage action spectra (Caldwell et al. 1986). This represents ambient summer conditions at this location (52.42°N, −4.07°E) (Appendix 1, b), but falls within the range currently experienced during the growing period in northern mid-latitudes (40–60°N) (Ries et al. 2000). However, it should be noted that glasshouses cannot accurately represent field conditions due to differences in UV-B:PAR, and UV-B:A ratios, and therefore, results should not be extrapolated to field conditions. Rather, UV-B has been applied to investigate the resource allocation during and after UV-B exposure, and the subsequent impact that this has upon reproductive success and phenology.

### Growth analysis

Total leaf area was assessed using a Delta-T area measurement system, controlled by a PC running the area recognition software Windias 2.0 (Delta-T devices, Cambridge, England). Roots were washed in running water to remove compost before root, leaf and stem material were dried separately at 60°C for 48 h. Specific leaf area (SLA, ratio of leaf area to leaf dry weight), leaf area ratio (LAR, ratio of leaf area to total plant dry weight), relative growth rate (RGR, rate of increase of total dry weight), and net assimilation rate (NAR, rate of dry weight production in relation to total leaf area) were calculated using standard formulae stated in Hunt et al. (2002). For RGR and NAR, plants were selectively paired by their dry weight ensuring that the smallest plant in each harvest was paired with the smallest plant in the subsequent harvest.

### Leaf epidermal morphology

At harvest 2, in a comparable method to Lake et al. (2009), three leaves were removed per plant, from three plants per treatment, and used to produce impressions of the adaxial and abaxial leaf surfaces. Due to treatment effects, it was impossible to be certain that chosen leaves were at the same developmental stage; instead, care was taken to choose mature leaves of approximately the same size and from similar positions on each plant. A strip of cellulose diacetate was softened with a drop of acetone for 20–30 sec before a leaf was pressed firmly onto this to create a leaf imprint. A cellulose varnish was applied to the imprint, and when dry, this was peeled off and mounted onto a slide. Slides were viewed at 400× magnification, and five fields of view of each leaf impression were assessed for stomatal and epidermal cell number. The area independent stomatal index was calculated as the ratio of stomatal cells to epidermal cells using formulae stated in Lake et al. (2009).

### Floristic characteristics and seed germination

After harvest 2, 20 plants from each treatment were fitted with aracon tubes (Betatech, Gent, Belgium) and left to flower. Reproductive measurements were begun on the date at which the first visible flower spike was observed in any plant. Following flower initiation, the presence of flower spikes and open flowers was assessed approximately every 2 days, while floral stem height and number of siliques were counted every 7 days for 7 weeks. When ripe, siliques were harvested from individual plants and the seed was weighed to find the total seed biomass per plant. For the germination assay, 25 seeds per plant were placed onto moist filter paper within replicated Petri dishes (*n* = 20). These were stratified at 4°C for 2 days before being transferred to a controlled growth cabinet (Vindon scientific, Rochdale, UK) in the dark at 22°C for the next 7 days. The number of germinated seeds over this period was used to calculate percentage germination.

### High-performance liquid chromatography (HPLC)

Leaves were analyzed for foliar phenolics and soluble sugars, to give an indication of metabolites induced for UV-B defense and as osmoprotectants in response to drought. Samples of freeze-dried leaf material (20 mg, *n* = 6) were ground and extracted twice in 70% methanol. The supernatant was dried to a pellet using a vacuum centrifuge (Savant SpeedVac SPD121P, Thermo-scientific, Asheville, USA) before re-suspension in 500 μL 70% methanol. A 250 μL aliquot was taken for analysis of sugars, with 50 μL added to 950 μL of a buffer comprising 5 μM H_2_SO_4_ with a 5 μM crotonic acid internal standard. Samples were analyzed for sugars using a Jasco HPLC system comprising a UV-1575 UV/Vis detector, LG-980–02 ternary gradient unit, PU-1580 HPLC pump, A-1555 intelligent sampler, RI-2031 RI detector, and CO-965 column oven (Jasco Ltd, Essex, UK). Sugars were identified based upon their retention time and comparison with an internal library of standard compounds.

A solid-phase extraction was performed on the remaining 250 μL sample using a C^18^ column (Sep-Pak vac 500 mg, Waters Ltd, Elstree, UK) before vacuum centrifugation of the sample to dryness. The dried pellet was suspended in 200 μL 100% methanol and analyzed using a waters HPLC system comprising an autosampler, Waters 600 Controller, Waters 996 photodiode array detector, and Empower chromatography software. Phenols were separated on a Waters C_18_ reversed-phase Nova-Pak cartridge (4.0 mm, 8.0 mm × 100 mm). The mobile phase consisted of 5% acetic acid (solvent A) and 100% methanol (solvent B) with a linear gradient from 0 to 70%, B in A, over 35 min. The injection volume was 25 μL. Peak integration was performed using the Waters chromatography manager software and the six main peaks found were analyzed.

### Liquid chromatography-mass spectrometry (LC-MS)

LC-MS was performed on representative samples from the HPLC analysis to provide identification of the six main peaks found (Appendix 2). This allowed separate analysis of hydroxycinnamic acids (HCAs) and flavonoids, to determine differences in response of these two compound groups to the treatments. A Thermo Finnigan LC-MS system was used (Thermo Electron Corporation, Waltham, USA) comprising a Finnigan Surveyor PDA Plus detector, a Finnigan LTQ linear ion trap with ESI source, and a Waters C_18_ reversed-phase Nova-Pak column (4 μm, 3.9 mm × 100 mm). The auto-sampler tray was kept at 5°C and the column temperature was maintained at 30°C. Injection volume was 10 μL, the detection wavelength was 240–400 nm and the flow rate was 1 mL/min, with 100 μL/min going to the mass spectrometer. The mobile phase consisted of purified water-formic acid (A; 100:0.1, v/v) and HPLC grade MeOH-formic acid (B; 100:0.1, v/v). The initial condition was A:B (5:95, v/v), and the percentage of B increased linearly to 50% over 45 min. Mass spectra were acquired in negative ionization mode, and compounds were identified by comparison of their molecular mass and fragmentation patterns with those reported for *A. thaliana* in the literature (Stobiecki et al. 2006; Ringli et al. 2008; Nishiyama et al. 2010).

### Statistical analyses

All analyses were performed using Minitab version 14 (Minitab inc. Coventry, UK). Two-way Factorial ANOVA designs were used to allow assessment of the effects of drought, UV-B and their interaction. Square root transformation was applied to the data, where necessary, to improve homogeneity of variance and normal distribution.

## Results

### Growth analysis

**Figure 3:**
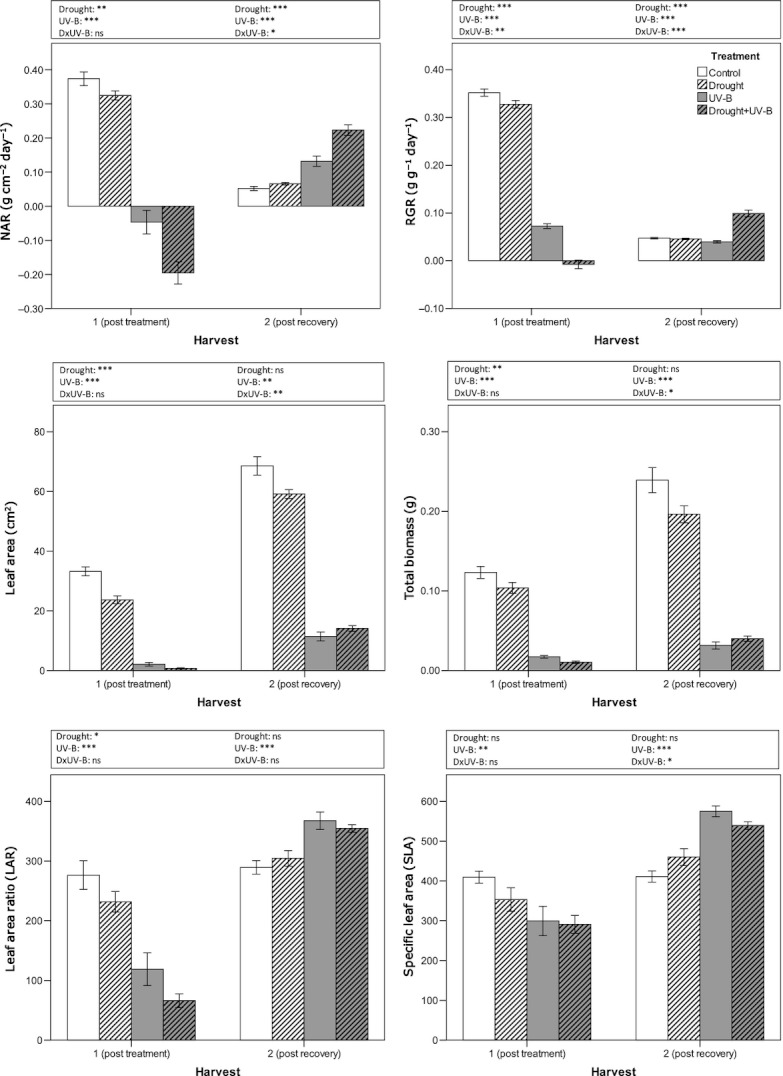
Effect of drought, UV-B and the combined treatments on growth parameters in *A. thaliana*. Harvests were taken following treatment (harvest 1), and following a 2-week recovery period (harvest 2). Significant differences show the result of a two-way factorial ANOVA to calculate the effects of UV-B, drought, and their interaction.

Both drought and UV-B caused significant reductions in rosette biomass, leaf area, and growth rate. Combined drought and UV-B had an interactive, negative effect on relative growth rate (RGR) following treatment (harvest 1) (Fig. 3). As a result, plants from the combined stress treatment had the lowest net assimilation rate (NAR), total dry weight, and leaf area at harvest 1. This represents an additive negative effect of the treatments at this time. Over recovery, however, the interactive effect of drought and UV-B was markedly different. Significant drought × UV-B interactions were observed in RGR and NAR over the recovery period, with values approximately two-fold higher in plants exposed to the combined treatments in comparison with all other treatments (Fig. 3). Consequently, plants exposed to the combined treatments recovered a rosette size comparable to UV-B-treated plants at this time point, despite the additive effects of drought and UV-B observed following treatment.

Both drought and UV-B caused reductions in leaf area ratio (LAR) following treatment, while UV-B also reduced specific leaf area (SLA). Drought had no effect on these parameters following recovery; however, significant increases were observed relative to controls in UV-B-treated plants. Of the two factors (drought and UV-B), UV-B had the greater impact upon growth. UV-B treatment resulted in a decreased rosette size, which was maintained throughout the remainder of the plant's lifecycle. Phenological assessment of rosette size and leaf number following recovery using the Boyes scale (Boyes et al. 2001) places the UV-B-treated plants as at an earlier growth stage (approximately principal growth stage 1.14–3.0) compared with control and drought-treated plants (principal growth stage 3.7–3.9). This suggests that UV-B delayed the developmental progression of the rosette.

### Leaf epidermal morphology

Measurement of leaf epidermal morphology was not possible at harvest 1 due to the small leaf size. Measurement following the recovery period (harvest 2) showed that stomatal number and stomatal index on both leaf surfaces were decreased as a result of UV-B treatment (Table 1). No drought effect or interaction was seen on either leaf surface, and epidermal cell number remained unaffected by any treatment.

**Table 1:** The effect of drought and UV-B on epidermal cell number (E), stomatal number (S), and stomatal index (Si) of the abaxial and adaxial leaf surfaces following a 14-day recovery period

	Mean (±SE)				*P*-value		
		
	Control	Drought	UV-B	D + UV-B	Drought	UV-B	D × UV-B
Abaxial							
E (mm^−2^)	1274.8 (65.4)	1370 (113)	1117.8 (54.9)	1220.3 (50.6)	0.202 ns	0.054 ns	0.964 ns
S (mm^−2^)	363.7 (20.6)	415.6 (44.3)	280.7 (14.5)	299.3 (17.9)	0.208 ns	0.001 **	0.544 ns
Si	22.196 (0.901)	22.810 (0.485)	20.096 (0.466)	19.032 (0.519)	0.720 ns	<0.001 ***	0.190 ns
Adaxial							
E (mm^−2^)	1682 (126)	1649 (170)	1504 (105)	1509 (69.4)	0.891 ns	0.227 ns	0.824 ns
S (mm^−2^)	435.6 (35.7)	418.5 (51.7)	317.04 (9.30)	351.1 (16.0)	0.811 ns	0.010 *	0.374 ns
Si	20.660 (0.321)	20.007 (0.503)	18.001 (0.631)	19.099 (0.482)	0.659 ns	0.002 **	0.093 ns

Values show the mean (SE). Results of a two-way ANOVA are shown with asterisks denoting significance (* *P* < 0.05, ** *P* < 0.01, *** *P* < 0.001).

### Foliar soluble phenolics and sugars

**Figure 4:**
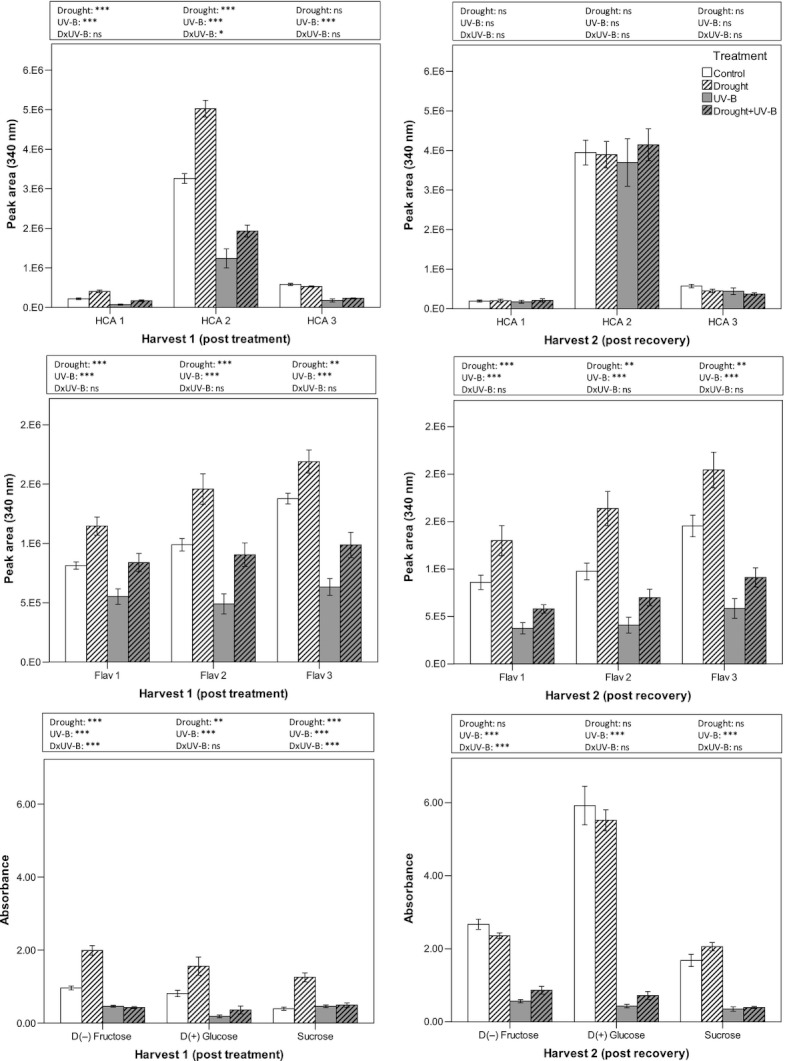
Effect of drought, UV-B, and the combined treatments on the foliar concentrations of Hydroxycinnamic acids (HCAs), Flavonoids (Flav), and sugars. Compounds were assessed from water/methanol extracts of leaf material using HPLC. Results of a two-way factorial ANOVA are also shown for each compound, to calculate the effects of UV-B, drought, and their interaction. Asterisks denote significance (* P < 0.05, ** P < 0.01, *** P < 0.001). Phenolics are expressed as their peak area integrated at 340nm per mg dry weight of material, while sugars are in units of absorbance. See Appendix 2 for tentative compound Ids of the phenolics.

HPLC identified six main phenolic compound peaks in the extracted leaf material, and comparison of their UV spectra separated three as hydroxycinnamic acids (HCAs), and three as flavonoids. LC/MS identified the HCAs as mainly sinapoyl compounds, with the most abundant being sinopoyl malate. The flavonoids were mainly Kaempferol glycosides (Appendix 2). Concentrations of HCAs and flavonoids were increased by drought, but reduced by UV-B over the treatment period (harvest 1), (Fig. 4). Over recovery, concentrations of HCAs in plants from each treatment returned to levels comparable to the controls. However, lower flavonoid concentrations were retained in UV-B-treated plants over the recovery period (Fig. 4).

Drought increased concentrations of Sucrose, D(+) Glucose, and D(-) Fructose following treatment, but concentrations were comparable to controls post recovery (Fig. 4). UV-B caused reductions in these compounds following treatment, and this was retained into the recovery period. The control and drought-treated plants showed a change in composition of sugars between treatment and recovery, with D(+) Glucose accumulating to approximately double the concentration of the other sugars (Fig. 4). This did not occur in UV-B-treated plants where all sugar concentrations remained in approximately equal proportions.

### Reproductive success

**Figure 5:**
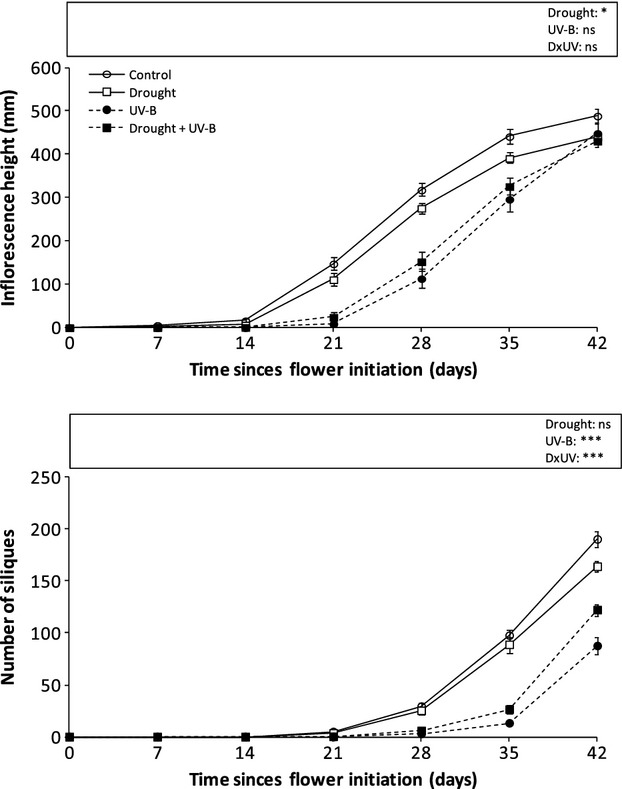
Inflorescence characteristics of *Arabidopsis thaliana* from each drought and UV-B exposure treatment combination. Time zero is the date at which the first plant produced a flower spike. The result of a two-way factorial ANOVA for the final time point is shown.

A significant UV-B effect and drought × UV-B interaction were seen in flower initiation (bolting), with approximate delays of 4, 14, and 11 days in the drought, UV-B and combined stress treatments, respectively (Table 2). These delays caused control and drought-treated plants to achieve a greater inflorescence height than UV-B and combined stress-treated plants over the majority of time points, while the inflorescence height of plants treated with combined stress was higher than plants treated with UV-B alone (Fig. 5). Flower opening was delayed by 8–9 days in UV-B and combined stress-treated plants relative to controls (Table 2). By the final week of measurements, however, all plants achieved a similar maximum inflorescence height, with only an approximate 10% reduction caused by drought (*P* = 0.049), (Fig. 5).

**Table 2:** Assessment of *A. thaliana* inflorescence characteristics. The date at which the first plant produced a flower spike was designated time zero. Date of flowering and date of first open flower represent the mean time (days) taken for flower initiation (bolting) and first flower opening relative to this. Total seed biomasses per plant and seed percentage germination were assessed at the end of flowering

			*P*-value		
			
	Treatment	Mean (SE)	Drought	UV-B	D × UV-B
Date of flowering	Control	07.68 (1.27)	0.653 ns	<0.001***	0.018 *
	Drought	11.25 (1.03)			
	UV-B	21.40 (1.28)			
	Drought + UV-B	18.95 (1.37)			
Date of first open flower	Control	18.13 (0.62)	0.370 ns	<0.001***	0.060 ns
	Drought	20.58 (0.74)			
	UV-B	26.88 (1.04)			
	Drought + UV-B	26.00 (1.02)			
Yield of seed (g)	Control	206.9 (9.82)	0.445 ns	<0.001***	0.518 ns
	Drought	190.5 (15.1)			
	UV-B	117.5 (11.7)			
	Drought + UV-B	116.1 (8.68)			
Percentage germination	Control	98.0 (0.616)	0.266 ns	<0.001***	0.924 ns
	Drought	96.6 (0.930)			
	UV-B	93.8 (1.100)			
	Drought + UV-B	91.2 (1.660)			

*P*-values show the result of a two-way balanced ANOVA.

*P < 0.05, **/ucodep* *P* < 0.001

Analysis of the final silique yield identified a significant drought × UV-B interaction, with plants from the drought, UV-B and combined stress treatments achieving silique yields of 86, 46, and 64% relative to controls (Fig. 5). No significant drought effect or drought × UV-B interaction was found in the total seed biomass per plant or percentage germination of seed. UV-B, however, caused a significant reduction in both parameters (Table 2). Despite this, all plants yielded seed, and the mean germination rate in each treatment was above 91%, resulting in plants from all treatments producing viable progeny.

## Discussion

This study provides an assessment of the interactive responses of *Arabidopsis thaliana* to a simulated episode of co-occurring UV-B and drought, typically observed during cloudless high atmospheric pressure weather events. Using a glasshouse facility, we particularly focused on treatment and interactive effects on plant phenology and reproductive success. Both drought and UV-B had negative effects on the growth of *A. thaliana*. The UV-B responses were likely more pronounced than would be expected in the field due to low PAR:UV-B ratio inherent in most glasshouse studies (Krizek 2004). In comparison, the drought treatment was relatively ‘mild’ when water was withheld from compost over a 14-day period. However, both treatments had adverse impacts on plant growth and the main focus of this study was to investigate recovery via phenology and seed production. Analysis overall revealed that *A. thaliana* exhibited substantial resilience to the treatment episode when considered to the end point of plant growth and seed production.

### Acclimation to UV-B

Acclimation to UV-B was observed via adjustment of leaf morphology in response to the UV-B treatment. Over treatment, UV-B caused a reduced specific leaf area (SLA), suggesting thicker leaves, a response that has been widely documented under UV-B irradiance, for example, Santos et al. (2004), Liu et al. (2005). Over recovery, both SLA and leaf area ratio (LAR) increased indicating investment of resources into maximizing the leaf assimilative surfaces, a similar response to that found in the ruderal *Dittrichia viscose* when treated with UV-B (Stephanou and Manetas 1997). With this came a concomitant increase in net assimilation rate (NAR), suggesting an improved productivity per unit of leaf area. Therefore, this adjustment of plant morphology may represent a mechanism to minimize leaf area, reducing UV-B damage during treatment, but maximize photosynthetic area and productivity over recovery.

Phenolic secondary metabolites are known to act as ‘sunscreens’ reducing UV-B damage (Landry et al. 1995; Bashandy et al. 2009) and their accumulation often occurs as part of the acclamatory mechanism to UV-B (Jansen et al. 2008). This protective response and capacity to induce such compounds builds up gradually in the field. However, plants grown in zero UV-B glasshouse conditions do not build such protection and/or the rapid capacity to respond to UV-B. This may explain why secondary metabolites were not induced in the current study and why adverse effects on plant growth were observed. Lake et al. (2009), using a similar UV-B regime, found a comparable initial reduction of growth in *A. thaliana* following UV-B exposure, with stimulation of flavonoids and recovery of growth indicative of UV-B acclimation only occurring following prolonged exposure. It is worth noting however, that *A. thaliana* grown under field conditions may show somewhat altered responses (e.g. Frenkel et al. 2008), for example initial foliar secondary metabolite concentrations are likely to be higher, providing increased inherent UV-B tolerance.

### Interaction with drought

Acclimation to drought in this study involved accumulation of both soluble phenolic metabolites (HCA's and flavonoids) and sugars. Comparable responses have previously been identified (Foyer et al. 1998; Rizhsky et al. 2004), and it is thought that sugars in particular act as osmoprotectants, limiting cellular water loss and therefore increasing drought tolerance (Hoekstra et al. 2001; Rizhsky et al. 2004). Following treatment, however, significant interactions were observed in concentrations of sucrose and fructose, whereby drought-induced accumulation of these compounds did not occur in plants also treated with UV-B. A concurrent interaction was seen in relative growth rate, with drought having a greater negative impact on plants also exposed to UV-B. One plausible mechanism for this response is that UV-B inhibited the accumulation of soluble sugars, preventing this acclamatory response to the drought treatment, and therefore caused a greater negative effect of drought in plants also treated with UV-B.

Previous investigation of drought and UV-B has also indicated that accumulation of osmolytes is an important determinant of interaction between these factors. For example, in cases where UV-B has been shown to reduce the negative impact of a concurrent drought stress, it is the accumulation of low molecular weight and soluble metabolites such as sugars which have been implicated (Schmidt et al. 2000; Alexieva et al. 2001). UV-B has previously been shown to have contrasting effects on concentrations of sugars depending on the species tested, and duration and intensity of UV-B exposure (Barsig and Malz 2000; Ghisi et al. 2002; Hilal et al. 2004). Therefore, the effect of UV-B on concentrations of these osmolytic compounds may be a key determinant of subsequent drought interactions, and the contrasting and species-specific responses of these metabolites to UV-B may be a source for the contrasting reports of interaction between drought and UV-B, for example, (Schmidt et al. 2000; Alexieva et al. 2001; Tian and Lei 2007; Sangtarash et al. 2009).

Significant interactions were also observed following 2 weeks' recovery from the experimental treatments. Paradoxically, it was the plants most negatively affected over treatment, which displayed the most rapid recovery of growth, with relative growth rate (RGR) almost doubled in plants exposed to combined stress compared with the other treatments. This recovery of growth may stem from a concurrent interactive effect on net assimilation rate (NAR), whereby plants treated with combined stresses attained higher NAR values than the other treatments. This demonstrates a significantly greater productivity per unit of leaf area (Hunt et al. 2002) in these plants, which probably contributed to their rapid recovery of growth. Further evidence for increased productivity can be seen in the significant interaction in fructose concentration at this time, with an increase in concentration in combined stress-treated plants relative to plants treated with UV-B alone. Such results may originate from an increased net photosynthetic rate, and future photosynthetic assessment may be beneficial for investigation of this interaction.

Recovery of growth following UV-B exposure is known to require scavenging of products of UV-B damage via antioxidant compounds including phenolic metabolites (Rozema et al. 1997). Flavonoid concentrations were significantly higher in combined stress-treated plants than those treated with UV-B alone, which may have contributed to their rapid recovery of growth. Furthermore, Rizhsky et al. (2004) have shown that *A. thaliana* exposed to combined drought and heat shock produced compounds not induced by single stresses; therefore, it may be that specific metabolites produced under the combined stress treatment aided re-growth over the recovery period. Although the source of the increased productivity and growth rate cannot be determined from the current study alone, it does highlight the potential for interactive effects to dramatically change over different stages of treatment and recovery, suggesting that single harvests for assessment of interactions may not be sufficient to determine the full scale of interactive responses.

The interactive effect of drought and UV-B on growth during recovery also had an important consequence for maintenance of reproductive success. During reproduction, earlier flower initiation in the combined stress treatment represented an interaction as these plants flowered sooner than those treated with UV-B alone. Furthermore, final yield of siliques was reduced by only 36% in the combined stress treatment relative to controls, while a 54% reduction was observed in plants treated with UV-B alone. Application of drought has previously been shown to reduce the negative impact of UV-B on yield in *Glycine max* (Sullivan and Teramura 1990), and in the current study, this is likely to be linked with the increased RGR and productivity observed in these plants over the recovery period, allowing faster recovery from stress and increased reproductive effort.

Total seed weight and percentage germination, however, were decreased by approximately 43 and 5% in both sets of UV-B-treated plants. These results suggest a synergistic effect of combined stress on floral timing and silique yield, but an overall reproductive success comparable to plants treated with UV-B alone. The difference in silique yield between the UV-B and combined stress treatments may indicate a differential effect on floral meristem production; however, further study is required to confirm this. Nevertheless, results demonstrate that even a relatively mild water deficit, as in the current study, was sufficient to exacerbate UV-B responses during treatment, and subsequently partially ameliorate negative effects on the timing of reproduction and silique yield. Future studies should investigate the specific mechanisms involved to better understand interactive responses, particularly in the recovery of NAR as observed in the current study.

### The cost of recovery: Delay in phenology

All plants recovered to reproduce; however, unsurprisingly, such acclimation and recovery following treatment had associated costs for plant development. Phenological assessment of the rosettes at harvest 2 (following recovery) using the Boyes scale (Boyes et al. 2001) categorizes the UV-B-treated plants as at an earlier phenological stage due to their lower rosette area and fewer mature rosette leaves. Furthermore, despite using leaves of approximately the same size, stomatal number and stomatal index were significantly lower in UV-B-treated plants. This agrees with a previous assessment of UV-B effects on leaf epidermal morphology (Lake et al. 2009). However, Hectors et al. (2010) have shown that UV-B does not affect stomatal index (SI) directly, but changes in SI can occur at different phenological stages. This may indicate that the leaves assessed from both UV-B treatments were at an earlier stage of development than controls in the current study; however, the direct effect of UV-B on stomatal index may warrant further investigation, given previous differences in the reported effect of UV-B on this parameter.

Foliar sugar concentrations of the UV-B and combined stress-treated plants after recovery were also more similar to the profile of sugars in controls from 14 days earlier. UV-B can affect carbohydrates directly, for example (Lindroth et al. 2000; Quaggiotti et al. 2004), and this probably explains the reduced sugar concentrations of UV-B-treated plants. However, changes in foliar carbohydrates have also been linked with changes in plant phenology, especially in preparation for flowering (Gibson 2000; Ohto et al. 2001). The substantial accumulation of glucose seen in control and drought-treated plants over recovery may represent a phenological change in preparation for flower production. The lack of this response in plants from both UV-B treatments, coupled with their smaller rosette size, provides further evidence that UV-B caused a delay in plant phenology.

A delay in phenology is confirmed via assessment of the date of flower emergence. Boyes et al. (2001) classify the date of flower emergence as the start of principal growth stage five while first open flower is seen as the start of growth stage six. Drought caused a delay of approximately 4 days in flower emergence, whereas UV-B and combined stress-treated plants were delayed by 14 and 11 days, respectively. Similarly, UV-B caused a significant delay in the date of first flower opening. This represents a stress-induced delay in the timing of reproduction, also shown in *A. thaliana* in response to phosphorous deficiency and shading (Dorn et al. 2000; Nord and Lynch 2008). It has been hypothesized that the delay in flower initiation may be a mechanism to maximize resources before flowering, allowing maximum reproductive success (Nord and Lynch 2008). In the current study, this may be the case, as, despite the delay, all plants reproduced successfully producing viable seed.

### Conclusions and further work

The responses in this study clearly demonstrate the capacity for drought and UV-B to interact, affecting aspects of plant growth, chemistry, phenology, and reproductive characteristics. A key finding is the potential for these interactions to change markedly between treatment and recovery, suggesting that interactive effects of treatments over the plants lifecycle may be substantially different from those measured directly following exposure. The cost of observed UV-B acclimation and recovery was a delay in developmental timings, possibly representing an adaptation to maximize resources before reproduction. Our results demonstrate that even a mild, co-occurring drought has the capacity to counteract some of the impacts of UV-B during reproductive development. It should also be emphasized that even though such glasshouse experiments have limitations, our study clearly highlights the value of, and the need for, future field experimentation on this topic. Such research should focus on the mechanisms involved in interactive effects on leaf stomatal characteristics, maintenance of net assimilation rate, and the timing of reproduction.

## Appendix 1

(a) Soil matric potential in the drought and control conditions over the experimental treatment period. (b) Daily UV-B_BE_ regime in relation to ambient clear sky summer conditions for this location (52.42 °N, -4.07 °E) as calculated using the ACD: tropospheric ultra-violet (TUV) model and weighted using the Caldwell plant damage action spectra (Caldwell et al. 1986).

## Appendix 2

Hydroxycinnamic acid (HCA) and flavonol conjugates (Flav) identified in leaf extracts of *A. thaliana*. (a) Example HPLC chromatogram of *A. thaliana* leaf material showing the six main peaks detected. (b) Tentative compound IDs obtained from UV light absorption spectra and LC-MS analysis.
